# Worldwide practices in pulmonary function test reporting after lung transplantation

**DOI:** 10.1016/j.jhlto.2025.100347

**Published:** 2025-07-16

**Authors:** Jens Gottlieb, Erika Lease, Merel Hellemons, Kieran Halloran, Tereza Martinu

**Affiliations:** a Department of Respiratory Medicine and Infectious Diseases, Hannover Medical School, Hannover, Germany; b German Center for Lung Research (DZL), Giessen, Germany; c Division of Pulmonary, Critical Care, and Sleep Medicine, University of Washington, Seattle, Washington; d Department of Respiratory Medicine, Erasmus University Medical Center Rotterdam, Rotterdam, the Netherlands; e Erasmus MC Transplant Institute, Erasmus University Medical Center Rotterdam, Rotterdam, the Netherlands; f Department of Medicine, University of Alberta, Edmonton, Canada; g Toronto Lung Transplant Program, University Health Network, Toronto, Ontario, Canada; h Division of Respirology, Department of Medicine, University of Toronto, Toronto, Ontario, Canada

**Keywords:** lung transplantation, respiratory function tests, reference values, forced expiratory volume, oscillometry

## Abstract

**Background:**

Spirometry interpretation after lung transplantation (LTx) is complex, requiring a clear understanding of reference values and normal thresholds globally. Abnormal values are typically based on general population reference standards.

**Methods:**

In April 2025, a 10-question online survey was conducted among International Society of Heart and Lung Transplantation-members to determine the reference values and normal thresholds used worldwide for post-transplant spirometry interpretation.

**Results:**

Responses from 58 centers, covering 22,080 lung transplant recipients and representing ∼40% of global transplant activity, were analyzed. Most patients (63%) were followed in the United States or Canada. Global lung initiative (GLI) reference was used for 60%, with 47% using the race-neutral version. The lower limit of normal (LLN) was reported in only 19% of pulmonary function assessments. Regional differences were noted in obstruction definition and impairment grading.

**Conclusions:**

While GLI-reference values are widely adopted, 40% of centers have yet to implement them, and LLN remains underutilized in spirometry reporting after LTx.

## Background

The interpretation of lung function using reference values is a fundamental aspect of respiratory medicine. Recently, race-neutral reference standards have been developed based on large, diverse population cohorts.^1^

The International Society of Heart and Lung Transplantation (ISHLT) has an established consensus definition for chronic lung allograft dysfunction, while ongoing efforts aim to formally define other phenotypes of graft dysfunction including baseline lung allograft dysfunction (BLAD). These classifications rely on spirometry.

Spirometry interpretation after lung transplantation (LTx) is complex,^2^ as abnormal values are common and assessed based on recipient rather than donor reference values. Retrospective studies have linked abnormally low forced expiratory volume in one second (FEV_1_) and forced vital capacity to poorer graft survival following both single and bilateral LTx.^3^, ^4^ Lung function interpretation post transplantation and definition of BLAD require a comprehensive understanding of the reference values and normal thresholds applied.

We conducted a survey to assess reference values and methods globally for postlung transplant pulmonary function testing (PFT).

## Methods

An online survey (SoSci Survey GmbH, Munich, Germany) was conducted among members of the ISHLT Pulmonary Professional Community via the "ISHLT Connect" platform between April 7 and May 2, 2025. In addition to questions regarding center activity and the number of patients in follow-up, the survey covered the use of reference values, classification of lung function impairment severity, definitions of abnormal findings, available PFT techniques, and documentation of the ISHLT baseline. The survey was also accessible to nonmembers and targeted pulmonologists involved in post-transplant follow-up care.

The survey included 10 questions, 8 of which were multiple-choice with single or multiple possible responses. Options for comments were also provided. Responses were considered valid if the questionnaire was completed through to the final page. Due to the anonymous nature of the survey and the absence of personal data collection, ethics approval was waived.

To assess the weighted distribution of selected options, the percentage of each response was multiplied by the number of active follow-up patients at each center (e.g., 50% selecting option A at a center with 250 patients in follow-up = 125 patients received option A). The proportion of each option was calculated within the total cohort in patients in follow-up in participating centers, thus reflecting the global utilization of each practice among lung transplant recipients.

Continuous variables were summarized using medians with interquartile ranges (IQRs), while categorical variables were summarized using counts with percentages. Categorical variables were compared using Fisher’s exact test. A *p*-value of 0.05 was considered significant.

## Results

The questionnaire was completed 59 times. One duplicate entry was excluded, resulting in 58 centers being included. Participating centers represented 20 countries across 6 continents: North America (*n* = 31), South America (*n* = 2), Europe (*n* = 22), Oceania (*n* = 1), and Asia (*n* = 2).

Twenty-two centers (38%) reported an annual volume of 20 to 49 lung transplants, 13 centers (22%) performed 50 to 99 transplants per year, and 10 centers (17%) reported performing 100 or more procedures annually. Altogether, the participating centers represented approximately 40% of the global LTx activity^5^ and a median of 64% of their respective national activity (IQR 45%-100%).^5^

The survey participating centers reported actively following a total of 22,080 LTx recipients having had at least 1 in-person visit within the past year. The median number of patients under active follow-up per center was 300 (IQR 98-585). The majority (63%) of patients were followed in the United States or Canada.

In 81% of patients in follow-up, PFT was performed in university-affiliated laboratories. In 4%, testing was conducted in community hospitals; and in 15%, both university and community settings were used. One pediatric hospital (0.2% of patients) also participated in the survey.

**Figure 1:**
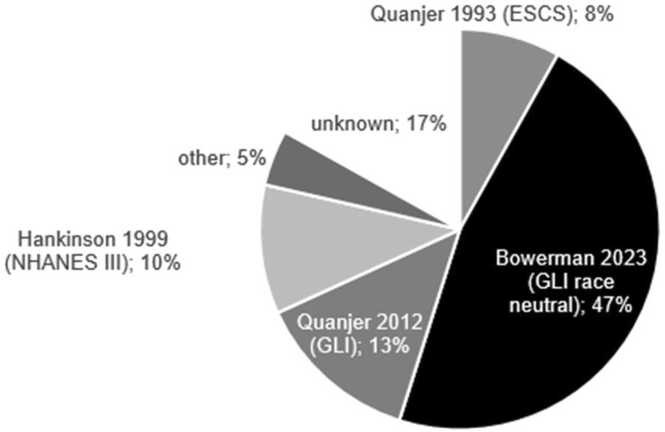
Global use of reference values based on the total number of lung transplant patients within participating centers (*n* = 22,080).

Overall, most LTx centers (63%) reported using global lung initiative (GLI) reference values, with 28 centers (44%) specifically using the race-neutral version. Weighted by the number of patients in follow-up, 60% of patients were assessed using GLI-reference values, and 47% by the race-neutral version (Figure 1). In 4 centers (7%), spirometry reports included multiple reference values.

In 19% of patients, the lower limit of normal (LLN) was mentioned in pulmonary function test reports—13% in North America versus 30% in others (*p* = 0.006). Similarly, the ISHLT-defined baseline was reported in 14% of cases overall (8% in North America vs 23% in other countries, *p* = 0.006).

In North America, severity of obstruction was significantly more often defined using the LLN for the FEV₁/forced vital capacity ratio. Outside of North America, the severity of spirometric impairment was more commonly graded using Z-scores (Table 1).

**Table 1:** Differences Between North America and Other Countries in Interpretation of Spirometry After Lung Transplantation

Definition of obstruction				
	Total (*n* = 22,080)	North America (*n* = 13,849)	Other countries (*n* = 8,231)	*p*-value
FEV1/FVC <0.7, *n* (%)	11,839 (54)	5,797 (43)	6,042 (73)	<0.001
FEV1/FVC < LLN, *n* (%)	8,191 (37)	6,202 (45)	1,989 (24)	0.002
Both FEV1/FVC <0.7 and LLN, *n* (%)	800 (4)	600 (4)	200 (2)	0.683
Raw data only, *n* (%)	950 (4)	950 (7)	-	0.014
Unknown, *n* (%)	300 (1)	300 (2)	-	0.498
Report of abnormal values^a^				
				*p*-value
Visual flagging, *n* (%)	3,562 (5)	1,562 (8)	2,000 (10)	0.08
Lower limit of normal, *n* (%)	11,877 (37)	10,215 (49)	1,662 (15)	<0.001
<80% predicted, *n* (%)	6,823 (21)	4,564 (22)	2,259 (20)	0.862
z-score, *n* (%)	7,463 (24)	3,359 (16)	4,104 (37)	0.001
Raw data only, *n* (%)	3,399 (11)	1,355 (7)	2,044 (18)	0.01
Unknown, *n* (%)	640 (2)	600 (3)	40 (0.4)	<0.001
Grading of abnormal values^a^				
				*p*-value
Raw data only, *n* (%)	4,890 (17)	1,562 (8)	3,328 (33)	<0.001
Descriptive only (mild, moderate, severe), *n* (%)	6,610 (23)	6,310 (31)	300 (3)	<0.001
Based on % predicted, *n* (%)	8,379 (29)	5,792 (31)	2,587 (22)	0.200
Based on z-score, *n* (%)	7,799 (27)	4,020 (22)	3,779 (38)	0.020
Unknown, *n* (%)	823 (3)	823 (8)	-	0.007

FEV_1_, forced expiratory volume in one second; FVC, forced vital capacity; LLN, lower limit of normal.

a Multiple answers were possible.

**Figure 2:**
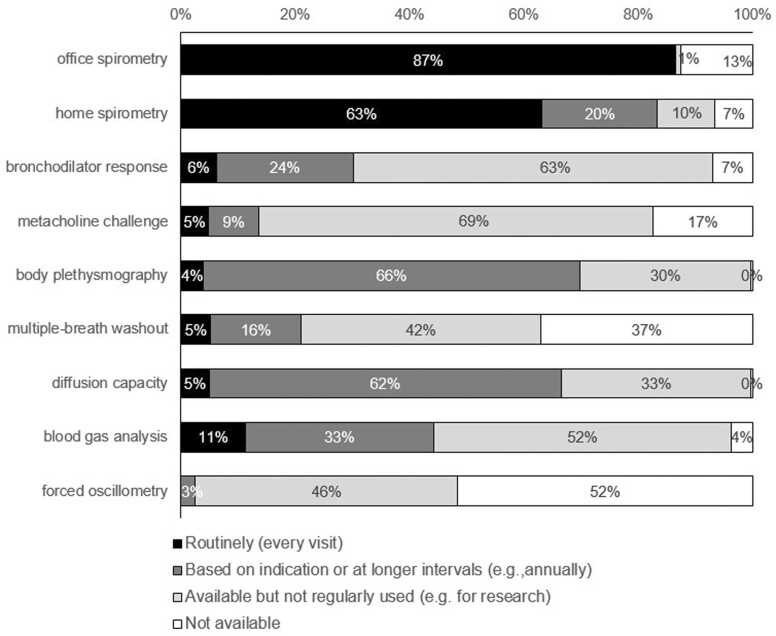
Global availability and use of various pulmonary function techniques in lung transplant centers based on the total number of patients (*n* = 22,080).

Home spirometry was used more frequently outside North America (73% vs 57%, *p* = 0.025). Body plethysmography (80% vs 63%, *p* = 0.012) and diffusing capacity measurements (76% vs 63%, *p* = 0.065) were more frequently used outside North America (Figure 2).

New techniques such as multiple-breath washout and oscillometry were available for 63% and 49% of follow-up patients, respectively.

## Discussion

This is the first systematic assessment of PFT in the follow-up of LTx recipients. The use of GLI equations predominates internationally, but there was a lack of uniformity in designating a normal threshold both within and between countries.

There is a general lack of publications addressing PFT globally. In a recent North American study,^6^ 26 out of 35 US centers (76%) similarly reported using GLI-reference values. The race-neutral GLI equations were also most frequently used. Reference value selection affects the diagnosis of abnormalities.^7^ Our results demonstrate substantial heterogeneity in PFT after LTx across the world, which will be an important point for harmonization moving forward. Differences in how abnormalities are reported—particularly the use of the LLN—may reflect long-standing regional practices, with the United States generally following the Global Initiative for Chronic Obstructive Lung Disease guidelines, while Europe tends to follow recommendations from the European Respiratory Society.

Our results are important for establishing a globally applicable and standardized definition of BLAD and for harmonizing future research efforts.

## Disclosure statement

The authors declare that they have no known competing financial interests or personal relationships that could have appeared to influence the work reported in this paper.

Charlotte Sybrecht, Hannover Medical School for her essential contributions in designing the Delphi surveys and analyzing the results.

## References

[1] Quanjer P.H., Stanojevic S., Cole T.J., Baur X., Hall G.L., Culver B.H. (2012). Multi-ethnic reference values for spirometry for the 3-95-yr age range: the global lung function 2012 equations. Eur Respir J.

[2] Kanj A.N., Scanlon P.D., Yadav H., Smith W.T., Herzog T.L., Bungum A. (2024). Application of global lung function initiative global spirometry reference equations across a large, multicenter pulmonary function lab population. Am J Respir Crit Care Med.

[3] Gerckens M., Mummler C., Richard A., Strodel J., Mertsch P., Milger K. (2024). Characterization of baseline lung allograft dysfunction in single lung transplant recipients. Transplantation.

[4] Paraskeva M.A., Borg B.M., Paul E., Fuller J., Westall G.P., Snell G.I. (2021). Abnormal one-year post-lung transplant spirometry is a significant predictor of increased mortality and chronic lung allograft dysfunction. J Heart Lung Transplant.

[5] Newsletter Transplant: International figures on donation and transplantation 2023 [Internet]. 2024. Available at: https://freepub.edqm.eu/publications/PUBSD-87/detail.

[6] Hachey K., Okelo S., Duncan F., Baugh A., Lovinsky-Desir S., Smith J.P. (2025). A National Survey of Spirometry Interpretation Practices. CHEST Pulmonary.

[7] Stanojevic S., Stocks J., Bountziouka V., Aurora P., Kirkby J., Bourke S. (2014). The impact of switching to the new global lung function initiative equations on spirometry results in the UK CF registry. J Cyst Fibros.

